# Windthrow disturbance impacts soil biogeochemistry and bacterial communities in a temperate forest

**DOI:** 10.1007/s11104-024-07086-8

**Published:** 2024-11-26

**Authors:** Bonnie G. Waring, Lena Lancastle, Thomas Bell, Martin I. Bidartondo, Pablo García-Díaz, Xavier Lambin, Elena Vanguelova, Francis A. Windram

**Affiliations:** 1 Georgina Mace Centre for the Living Planet, Department of Life Sciences, Imperial College, London, SL5 7PY England; 2 Ecosystem Stewardship, Royal Botanic Gardens, Kew, Richmond, TW9 3DS England; 3 School of Biological Sciences, University of Aberdeen, Aberdeen, AB24 3FX Scotland; 4 Present Address: Instituto de Ecología Regional (UNT-CONICET), Tucumán, Argentina; 5 Forest Research, Alice Holt Lodge, Farnham, GU10 4LH England

**Keywords:** Carbon cycling, Disturbance, Roots, Soil bacteria, Windthrow

## Abstract

**Aims:**

Forests across the world are subject to disturbance via wind, wildfire, and pest and disease outbreaks. Yet we still have an incomplete understanding of how these stressors impact forest biota—particularly the soil microbes, which govern forest carbon and nutrient cycling.

**Methods:**

Here, we investigated the impact of a severe windstorm on soil bacterial communities in Kielder Forest, a temperate coniferous forest in the north of England. Within ten individual sites, defined by common stand composition and topography, we established 50 m^2^ plots in undisturbed stands, and in nearby stands that were moderately and/or severely disturbed by windthrow. Soils were sampled within each of the 22 study plots, and analysed for changes in carbon and nitrogen content, pH, root biomass, and bacterial community structure. We separately sequenced bacteria from bulk soils, rhizosphere soils, and root tissues to assess whether disturbance impacts varied based on the proximity of microbiota to tree roots.

**Results:**

Less than a year after the storm, we found that the most severely disturbed stands had lower canopy cover, lower soil carbon content, higher soil pH, and a smaller fine root biomass than the undisturbed stands. Disturbance also impacted bacterial community beta-diversity, but the effects were subtle and did not vary among assemblages in bulk vs. rhizosphere soils.

**Conclusions:**

Impacts of aboveground disturbance on soil biogeochemistry can be significant, but soil bacterial communities are relatively well-buffered against these changes. However, altered patterns of root growth and carbon cycling may have longer-term implications for forest recovery after windthrow disturbances.

**Supplementary Information:**

The online version contains supplementary material available at 10.1007/s11104-024-07086-8.

## Introduction

Globally, forests play a critical role in sequestering carbon away from the atmosphere and stabilising the planet’s climate. However, the forest carbon sink is threatened by increasing frequency and intensity of disturbances, mainly driven by fire, pest outbreaks, and wind (Seidl et al. 2014; McDowell, et al. 1979), compounded by a decline in ecosystem resilience to disturbance (Forzieri et al. 2022). Moreover, drivers of disturbance (e.g. wildfire, drought, insects, and pathogens) often amplify one another’s effects (Seidl, et al. 2017). The pathway of forest recovery from disturbance is modified by the species composition and demography of forest communities (Anderson-Teixeira et al. 2013). To predict forest dynamics and carbon uptake in an increasingly unstable climate, we require a better understanding of how disturbances impact the forest biota that mediate carbon cycling.

In Europe, where forest disturbance has exhibited a pronounced increase over the past century, wind accounts for nearly half of observed forest damage (Patacca et al. 2023). Wind-driven disturbances range in intensity, from localised impacts which create small canopy gaps, to stand-replacing events. In turn, the scale and severity of disturbance affects forest demography, community dynamics, and ultimately aboveground carbon storage (Ulanova 2000; Mitchell 2013). Wind also has a strong influence on soil development: uprooted trees create pit-mound microsites that can persist for hundreds of years, amplifying the spatial heterogeneity of forest soils (Šamonil et al. 2010). A recent meta-analysis of forest disturbance effects on soils found that windthrow events trigger substantial losses of carbon from organic horizons (Mayer et al. 2023). This suggests that although windthrow events enhance organic matter inputs to soils in the short term, these effects are usually overwhelmed by longer-term losses. These losses can occur through direct physical disturbance (soil turbation and erosion) (Mayer et al. 2020), or can be microbe-mediated through increased decomposition, respiration and dissolved organic matter leaching. Carbon inputs may further be reduced in the long term via lags in the recovery of forest productivity, especially in the high-latitude/high-elevation forests where trees grow slowly (Mayer et al. 2023), and/or by post-disturbance forest management (e.g. salvage logging). Thus, the impact of wind on the total forest carbon sink is dependent upon the attributes of the affected forest, the scale and severity of damage incurred, and the time elapsed since the disturbance event.

Belowground responses that occur in the very short term (i.e., months to years after a disturbance event) represent a substantial knowledge gap in our understanding of windthrow impacts. These immediate changes in soil conditions are significant because they may influence the trajectory of forest recovery. The spatial heterogeneity in soil properties induced by a windthrow event influences the composition of regenerating vegetation (Fischer et al. 2002), and feedbacks between plant and microbial communities will play a vital role in determining successional dynamics (Reynolds et al. 2003). Changes in soil nutrient or water content post-disturbance might also impact subsequent seedling recruitment. For example, in one Scottish forest, soil disturbance induced by stump harvesting significantly reduced the growth and survival of tree seedlings, potentially due to changes in soil moisture regime (Vanguelova et al. 2017).

Very little is known, however, about disturbance impacts on soil microbial communities, and how these vary through space or over time. Chronosequences capturing several centuries of plant community succession reveal consistent shifts in microbial community composition as stands mature (Bai et al. 2021; Shao et al. 2019). We do not know whether the same successional processes play out at smaller and fragmented scales (i.e. within patches of forest disturbance), nor can we predict how long it would take for storm-impacted microbial communities to again resemble those in nearby undisturbed patches. Microbial biomass tends to decline steeply following wildfires, tree harvest, and storms, and may take several decades to rebound (Holden and Treseder 2013), suggesting compositional recovery may exhibit similarly long time lags. One study found a decrease in temperate forest soil bacterial diversity 80 years after a severe disturbance (wildfire) (Bowd et al. 2022), while another found an *increase* in diversity after clear-cutting 4–8 decades previously (Osburn 2019). However, soil assemblages in temperate forests are also very dynamic on sub-annual timescales, demonstrating predictable seasonal trends (López-Mondéjar et al. 2015), raising the possibility that communities might recover more rapidly from milder or more localized disturbance.

Storm-induced damage to trees will affect soil microbial (bacterial) communities in the short term via three mechanisms: physical disruption of soil structure, changes in the inputs of senesced plant litter (most notably, via a pulse of litterfall immediately following the disturbance), and changes in the growth of living roots. The extent to which each of these phenomena impact community structure will depend upon the location of the bacterial community (root-associated, rhizosphere, or bulk soil) and the severity of the forest disturbance. Thus, we might expect severe disturbances causing tree uprooting to impact the entire belowground community, while milder disturbances might only affect the bacteria which directly associate with tree roots. By investigating bacterial community shifts along gradients of wind damage intensity and proximity to roots, our goal was to shed light on the mechanisms by which aboveground disturbances influence belowground community dynamics in the short term.

We sampled soils from individual stands within planted temperate coniferous forests that were undisturbed, moderately, or severely disturbed by wind storms in the winter of 2021–2022. We characterized physicochemical changes in the soils and assessed compositional responses of bacterial communities in bulk soils, in the rhizosphere, and in association with the tissue of living plant roots. Our sampling design enabled us to test three predictions. First, we hypothesized that disturbance would impact soil abiotic properties (pH, C and N content) and root biomass through the aforementioned mechanisms (physical disruption, change in litterfall, and altered plant growth patterns). Second, we predicted that these soil changes would induce shifts in the taxonomic composition and diversity of bacterial communities. Third, we hypothesized that compositional shifts would depend upon the interaction between community type (bulk soil, rhizosphere, root-associated) and disturbance intensity, with higher sensitivity of plant-associated bacteria to milder disturbances, which might not result in physical disturbance of the soil profile.

## Methods

### Field sampling

Fieldwork was conducted at Kielder Forest, Northumberland, England (55.24N, 2.61 W; elevation 150 – 400 m). The site is dominated by planted Sitka spruce (*Picea sitchensis*), is underlain by peats (generally < 50 cm depth) over limestone, and receives ~ 1,300 mm of precipitation a year. The site has high windthrow risk due to its elevation and topographic position (Mcintosh ’ n.d.). Kielder Forest was impacted by several major storms over the winter of 2021–2022, notably Storm Arwen (November 2021), and later Storms Dudley, Eunice and Franklin (February 2022) (see: https://www.metoffice.gov.uk/weather/learn-about/past-uk-weather-events). Initial post-storm surveys revealed large areas of snapped and windthrown trees.

In spring 2022,10 sites were identified within Kielder Forest, with each site defined by homogenous stand composition, topography, and soil conditions, but spatially varying storm impacts. Using preliminary survey data on the spatial distribution of windthrown sites in Kielder Forest collected by Forestry England and the National Forest Estate Sub-compartments England 2019, we started by selecting all sites larger than one hectare and dominated by Sitka spruce. Subsequently, we used statistical matching (Huntington-Klein 2022) to define a set of candidate pairs of undisturbed plots and windthrown plots within each site. Statistical matching uses the similarity, quantified by a multivariate distance, between two or more points (undisturbed and windthrown plots in our case) based on their characteristics (e.g., stand composition, topography, and soil conditions). The lower the distance score, the more similar the characteristics of any given combination of candidate pairs of undisturbed and windthrown plots. We used the R package *StatMatch* (D’Orazio and ‘StatMatch’. 2022) to estimate the Mahalanobis distance between all potential pairs of undisturbed and windthrown plots within Kielder Forest. We matched plot pairs on the following eight variables: (i) total area of the undisturbed and windthrown areas (ha); (ii) cover of conifer species (%); (iii) richness of conifer species (number of species); (iv) elevation (masl); (v) soil root conditions; (vi) soil nutrients; and (vii) soil oxygen availability, with data for (v) through (vii) from FAO’s Harmonized World Soil Database v 1.2. All variables were centred and scaled prior to matching. After matching all potential combinations of undisturbed and windthrown plot (116,250 combinations), we chose those 14 pairs with both the lowest Mahalonobis distance and the lowest geographical distance between windthrown and matched undisturbed plots. We then refined our candidate pool of paired plots in the field. We considered the accessibility of the sites, the presence of windthrown areas not previously mapped within our chosen undisturbed sites, and consulted with Forestry England to secure access and ensure that the selected plots would not be harvested or salvaged during our fieldwork. In the end, we identified ten experimental sites, each of which encompassed two closely located, paired plots which were similar in all characteristics except the presence of wind damage.

**Fig. 1:**
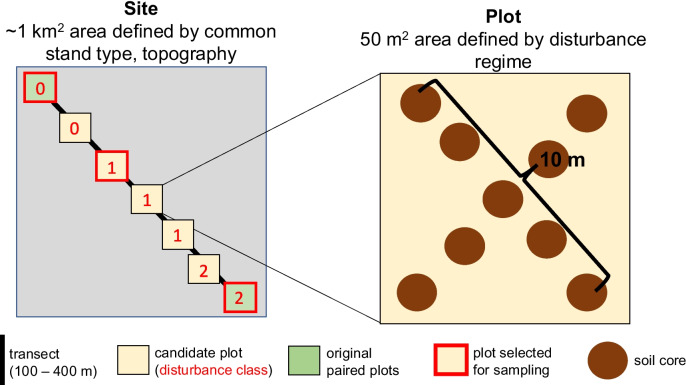
Schematic of the sampling design. Within each of 10 unique sites (defined by a common stand type and topography), we established two paired plots (50 m^2^) which were similar in all aspects except their degree of storm disturbance. A transect was laid between these two paired plots, with storm disturbance intensity (0 – undisturbed, 1 – moderately disturbed, 2 – severely disturbed) scored at regular intervals. From this transect, we selected a maximum of three plots for soil core sampling, one in each disturbance class. Note that some transects only yielded plots in two of the classes (0 and 1 or 0 and 2), and therefore not all sites contained 3 plots

The procedure described above yielded two plots per site, based upon a binary metric of windstorm damage (disturbed/undisturbed). However, we wished to capture varying degrees of windthrow disturbance within our experimental design. To do this, we laid a transect (minimum 100 m, maximum 400 m) between the paired plots (undisturbed, windthrown) nested within each site, and assessed storm impact at observation points every 20 m along this transect. To this end, we devised a categorical index of windthrow damage, based on the 10 trees nearest to the observation point: undisturbed (no fallen/snapped trees), moderately disturbed (50% of trees snapped or fallen, with no soil disturbance), and severely disturbed (50% of trees snapped or fallen, with formation of soil ‘pits’ and ‘mounds’ due to tree uprooting). We then randomly selected observation points belonging to each disturbance class upon which to centre 50m^2^ soil sampling plots (Fig. 1).

The end result of our sampling regime was a set of 22 plots nested within ten sites, such that each site contained one undisturbed plot and at least one additional plot in a moderate or severe disturbance class. Seven sites contained a severely disturbed plot, one site contained moderately disturbed plot, and two sites contained three plots (one for each disturbance class). Thus, we surveyed 22 plots in total across the 10 sites (10 undisturbed, 3 moderately disturbed, and 9 severely disturbed).

Beginning on 15th August 2022, within each plot, total number of trees was noted and all tree species were identified, recording how many were fallen or snapped, as well as the depth of soil pits induced by windthrow (if present). Hemispherical photos were taken to assess canopy cover at the centre of each plot, and subsequently analysed to determine canopy cover Leaf Area Index (LAI, or the ratio of leaf to ground area) with Cano.py, a new parallelised hemispherical photography and analysis pipeline (see Appendix 1 for details). Then, two perpendicular 10 m transects were placed diagonally across each plot, intersecting at the midpoint. At the intersection and then every 2.5 m along these transects, we used a soil corer (Dutch auger) to take a bulk soil sample to 20 cm depth. Additionally, immediately adjacent to the auger hole, we extracted volumetric root samples (Powers et al. 2005). A knife and trowel were used to excavate a 10cmx10cmx20cm ‘root block’ (unlike a soil probe or auger, this technique avoided compressing the soil), which was gently shaken to remove bulk soil while retaining all roots and their associated soil. With 22 plots across 10 sites, and 9 soil cores and 9 root blocks per plot, we obtained 396 samples in total. Samples were stored on ice in coolers to be transported back to the laboratory within 24 h.

### Laboratory processing

Small subsamples of each bulk soil core were immediately stored in a −80 C freezer for subsequent soil microbiological analysis, with the remainder allowed to air dry at room temperature for subsequent chemical analysis. Rhizosphere soil was extracted from the root blocks by gently brushing the adhering soil off roots into a sterile sample container; subsamples of these soils were also then placed into −80 C storage, with the remainder air-dried for chemical analysis. The roots themselves were then divided into two portions: one portion was air-dried, rinsed gently with drops of distilled water to remove soil, oven-dried and then weighed, and the other portion was weighed wet, and then stored in 80% ethanol for subsequent DNA extraction. We determined gravimetric moisture content of a small subsample of roots from each plot in order to convert the wet weight of the ethanol-preserved roots to a dry weight. Dry masses of both root portions were summed to calculate total root mass in each sampled 2000 cm^3^ root block.

### Soil biogeochemical measurements

pH was determined on bulk and rhizosphere soil slurries in a 1:5 ratio with distilled water. Soil samples were then ground to a fine powder, acidified with addition of 6% H_2_SO_3_ to remove carbonates, oven-dried at 65 C, and analysed for total organic carbon and nitrogen content on a ThermoFlash EA 1112 Elemental Analyser.

### Microbial compositional analysis

We used a Qiagen DNeasy PowerSoil Pro kit (Qiagen, Hilden, Germany) to extract DNA from bulk soils (those extracted with an auger), rhizosphere soils (those shaken from roots), and preserved roots themselves. Prior to extraction, individual core samples from each plot were bulked to yield 22 unique samples for each community type (bulk soil, rhizosphere, root-associated; 66 DNA extractions in total). We amplified the V4 region of the bacterial 16S rRNA gene using the primers 515F-806R (Caporaso et al. 2011) with a 3 min denaturation at 94 C for 3 min, followed by 35 cycles of 94 C (45 s), 50 C (60 s), and 72 C (90 s), concluding with a 10 min extension at 72 C. Amplicons were cleaned with AMPure XP SPRI beads (Beckman Coulter, Indianapolis, IN, USA) and submitted to the Earlham Institute (Norwich, UK) for index PCR, library quantification and normalization, and sequencing on the MiSeq platform. Sequences were demultiplexed, paired, and analysed using the QIIME2 bioinformatic pipeline (Caporaso et al. 2010), with denoising and chimera removal performed with DADA2 (Callahan et al. 2016), and taxonomy assignment of unique Amplicon Sequence Variants (ASVs) through the ‘classify-consensus-blast’ command. We obtained a median of 1,563,592 sequence reads per sample (range: 23,254 – 1,976,368), which declined to an average of 851,016 reads per sample after denoising (range: 17,327 – 1,458,483). Non-bacterial sequences were filtered from the resulting ASV table, and all samples were rarefied to 20,000 individual sequences/sample (excluding one sample with < 20,000 reads) prior to downstream analysis.

## Statistical analysis

To verify that our categorical disturbance index was valid, we analysed the number of intact, snapped, and fallen trees in each category with a Poisson regression model. We also used a one-way ANOVA to analyse canopy cover as a function of disturbance class.

### Test of Hypothesis 1: disturbance impacts on soil physicochemical properties

We used two-way ANOVAs to assess soil pH, C content, N content, and root biomass as a function of disturbance class and site identity. To yield a conservative estimate of effect sizes and avoid inflating degrees of freedom, we averaged all biogeochemical data measured within a plot, treating the 9 soil cores within that plot as analytical replicates rather than true statistical replicates. To explore whether disturbance responses varied among sites, we calculated log response ratios for each site as: $LRR=ln\frac{{X}_{D}}{{X}_{C}}$, where X_D_ represents a given response variable (e.g. soil carbon) in a disturbed plot, and X_C_ is the response variable for the control (undisturbed) plot in the same site.

### Test of Hypothesis 2 and 3: disturbance impacts on bacterial communities as a function of their proximity to plant roots

We used an ANOVA to assess bacterial alpha diversity (ASV richness, evenness [McIntosh’s evenness measure], and diversity [Shannon index]) patterns in relation to community type (bulk, rhizosphere, root-associated), disturbance class, and site. We then assessed compositional variance (i.e. species turnover or beta diversity) in response to the same factors, using a permuational ANOVA (*vegan* package (Oksanen, et al. 2022)), visualising the results with an non-metric multidimensional scaling (NMDS) plot using a Bray–Curtis dissimilarity matrix. We partitioned the variance associated with the spatial (latitude/longitude) and environmental (soil pH, C, N; canopy cover) components of site identity. To assess taxonomic patterns underlying compositional responses to treatments, we used the Dirichlet-Multinomial parameter test comparison in the *HMP* package (Rosa, et al. 2019) to test whether the distribution of ASVs into different bacterial phyla varied with disturbance class or community type. Finally, we conducted ANalysis Of COMposition (ANCOM) tests in QIIME2 to identify which ASVs exhibited significant variation in abundance among community types or disturbance classes. We rarefied communities to 1,000 sequences per sample prior to conducting ANCOMs; therefore, these analyses largely focus on the most abundant taxa.

## Results

Our biogeochemical and soil bacterial survey of storm-impacted forest stands revealed that windthrow disturbance was superimposed over substantial background variation in belowground properties. As expected for a peaty soil, all soil samples exhibited relatively high C content (23.0 ± 2.1% [SE]), low N content (1.07 ±0.09%), high C:N ratios (20.9 ± 0.6), and low pH (3.75 ± 0.07). Root biomass ranged from 0.50 to 3.11 mg cm^−3^, confirming that fine roots represent a substantial belowground carbon stock (25 to 156 kg ha^−1^ in the top 20 cm, assuming 50% of dry root mass is carbon). Spatial variation was substantial, with soil C content varying nearly eightfold, soil pH by about 1.3-fold, and root biomass over twofold among the ten sites we studied. Moreover, in these forest soils, bacterial communities were highly diverse, with an average of 2,647 ASVs observed in each sample (post-rarefaction to 20,000 sequences per sample). As was true for soil biogeochemical parameters, diversity patterns also exhibited pronounced variability across the landscape, with mean ASV richness ranging from 1953 to 3424 among sites.

### Impacts of disturbance on soil biogeochemistry (Hypothesis 1)

**Fig. 2:**
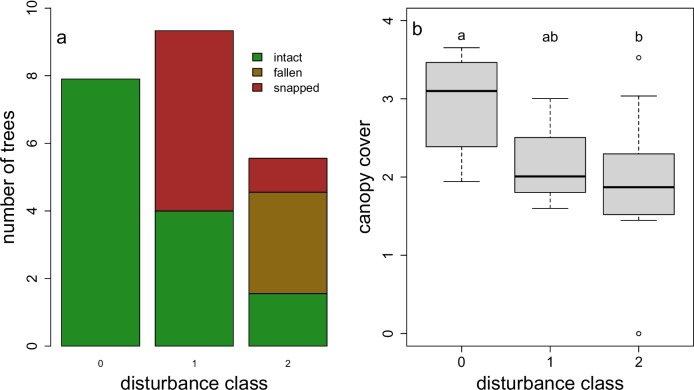
**a** Mean number of intact, fallen, and snapped trees in each disturbance class, as assessed every 20 m along ~ 200 m transects. **b.** Mean canopy cover (LAI) of study plots in each disturbance class. Letters indicate significant differences among categories in a Tukey’s HSD post hoc test

Our categorical disturbance index was a good metric of windthrow damage, with significantly more intact trees in the undisturbed plots than the moderately disturbed (*z* = −2.20, *P* = 0.028) or severely disturbed plots (*z* = −5.60, *P* < 0.001, Fig. 2a). Additionally, canopy cover within each plot varied significantly among disturbance classes (F_2,19_ = 3.92, *P* = 0.038, Fig. 2b): LAI decreased from 2.94 in undisturbed plots to 1.92 in the most severely disturbed plots.

**Fig. 3:**
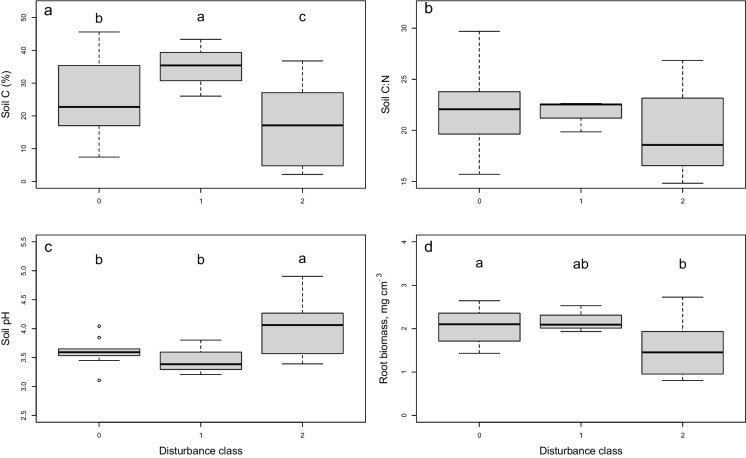
Variation in soil parameters (**a.** soil carbon, **b.** soil C:N ratio, **c.** soil pH and **d.** root biomass) by disturbance class across 22 forest plots (N = 10 undisturbed (disturbance class 0), three moderately disturbed (disturbance class 1), and nine severely disturbed (disturbance class 2). Letters indicate significant differences among categories in a Tukey’s HSD post hoc test

Soil C content, soil pH, and root biomass all varied significantly among disturbance classes, and most parameters also varied across sites (Table 1**, **Fig. 3). In comparison with undisturbed plots, severely disturbed plots had 34% lower soil C contents, 11% higher pH, and 26% smaller root stocks. Undisturbed and moderately disturbed plots usually did not differ from each other, except for soil C content, which was highest in the intermediate disturbance class.

**Table 1:** Results of two-way ANOVAs examining effects of windthrow disturbance and site identity on soil biogeochemical parameters. F statistics are shown, with *P* values in parentheses. (Residual df = 10)

	Df	Soil C (%)	Soil C:N	Soil pH	Root biomass (mg cm^−3^)
Disturbance class	2	14.79 (0.001)	2.14 (0.168)	6.81 (0.014)	4.25 (0.046)
Site	9	10.68 (< 0.001)	3.61 (0.029)	2.89 (0.057)	2.04 (0.140)

### Bacterial community responses to disturbance (Hypothesis 2) as a function of community type (Hypothesis 3)

**Fig. 4:**
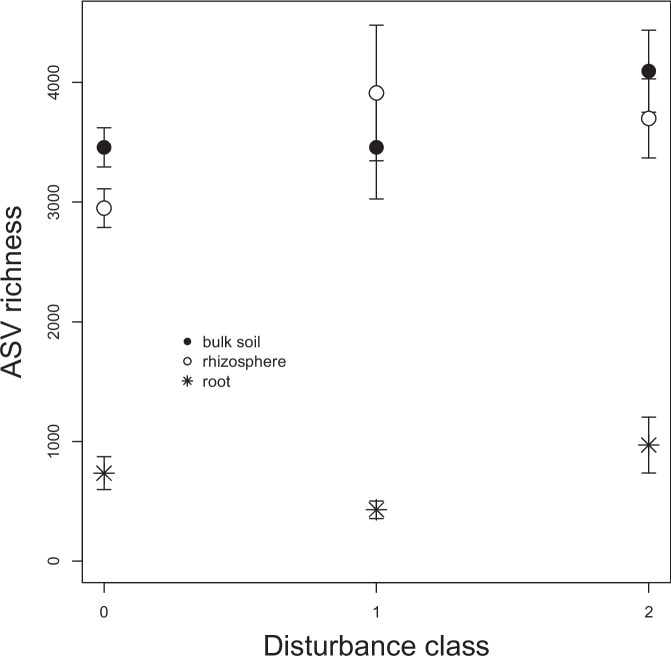
Variation in ASV richness among bacterial community types (bulk soil, rhizosphere soil, root-associated) and disturbance classes (0 = undisturbed, 1 = moderately disturbed, 2 = severely disturbed)

**Fig. 5:**
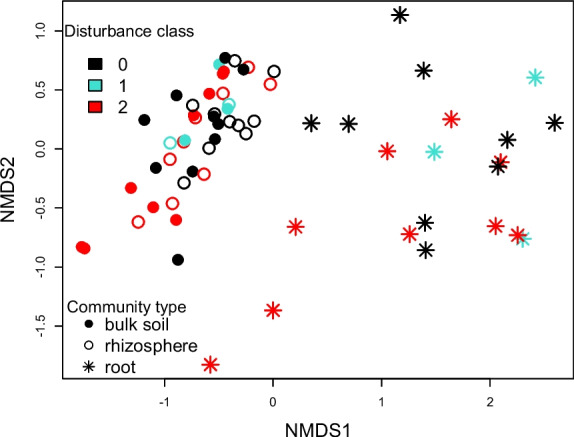
NMDS ordination showing variation in bacterial community composition as a function of disturbance class and community type

**Fig. 6:**
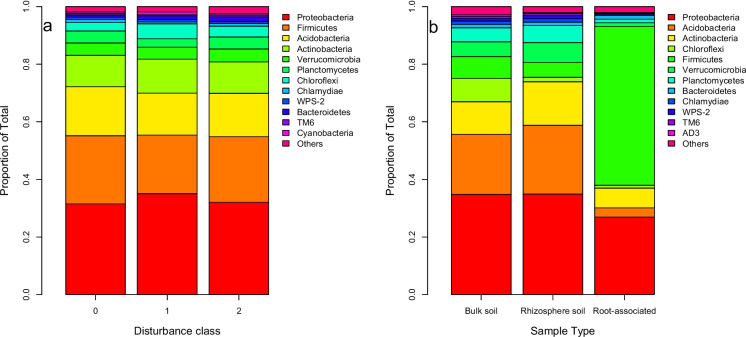
Distribution of bacterial phyla ASVs in relation to **a.** disturbance class and **b.** community type

Alpha diversity (i.e., number of unique ASVs per sample) varied significantly according to community type (F_2,46_ = 201.0, *P* < 0.001), disturbance score (F_2,46_ = 7.4, *P* = 0.002), and site (F_9,46_ = 6.8, *P* < 0.001); however, the Shannon diversity index and McIntosh evenness index did not vary among disturbance categories (*P* > 0.05). Rhizosphere and bulk soils exhibited five-fold greater ASV richness than root-associated communities, and diversity increased with disturbance regardless of the community type examined (Fig. 4). Community composition (beta diversity) also responded to disturbance, although the influence of community type was much stronger (Table 2), explaining ~ 20% of the variance whereas disturbance only explained ~ 4%. Communities in severely disturbed plots tended to differ from those in undisturbed or moderately disturbed plots (Fig. 5), although the distribution of taxa among phyla did not vary according to disturbance class (X_dc_ = −133.9, *P* = 0.999, Dirichlet-Multinomial test, Fig. 6a). In the ANCOM analysis, none of the most common ASVs were differentially distributed among disturbance categories, either. Meanwhile, root-associated bacteria were markedly compositionally distinct from those dwelling in the soil (Fig. 5). The compositional variation observed among community types could be detected at the phylum level (X_dc_ = 432.3, *P* < 0.001, Fig. 6b): in root-associated communities, Firmicutes were substantially more abundant, and Actinobacteria and Acidobacteria were less abundant. Moreover, twenty-six of the most common ASVs were found to be differentially distributed among community types in the ANCOM analysis. Eight ASVs in the actinobacterial orders Acidobacteriales, Ellin6513, Solibacterales, and Acidimicrobiales were significantly more abundant in bulk and rhizosphere soil than on root tips, and the same was true for three ASVs in the actinobacterial order Actinomycetales and three alpha-proteobacteria in the order Rhizobiales. Meanwhile, seven ASVs in the Firmicutes genera *Bacilllus, Cohnella, Paenibacillus,* and *Sporosarcina* were much more abundant on roots, as was also true for a gamma-proteobacterial taxon in genus *Enhydrobacter.*

**Table 2:** Results of a PERMANOVA examining impacts of windthrow disturbance, bacterial community type (bulk soil, rhizosphere soil, root-associated), and site identity on beta diversity

Factor	df	F value	P value	R^2^
Disturbance class	2	1.72	0.011	0.039
Community type	2	8.54	0.001	0.195
Site	9	1.94	0.001	0.199
Disturbance x community type	4	0.95	0.559	0.043

Site identity explained 20% of the variance in bacterial community composition, regardless of community type (Table 2). We conducted a variance partitioning analysis to further explore this pattern, quantifying variation due to space (i.e., site location) and environmental variables measured at the plot scale (canopy cover, soil carbon and nitrogen, soil pH, and root biomass), all of which varied strongly by site (Table 1). These factors in total explained only 6% of the observed variance in bacterial communities, with environmental variables accounting for 5.4%, and space (and the covariation between environment and space) explaining less than 1% of the data overall. A PERMANOVA examining community variation according to the above-mentioned factors revealed that soil carbon (F_1,56_ = 3.37, *P* = 0.001), nitrogen content (F_1,56_ = 2.06, *P* = 0.012), and root biomass (F_1,56_ = 1.55, *P* = 0.047) were significant predictors of beta-diversity, while the effect of pH (F_1,56_ = 1.50, *P* = 0.077) was non-significant.

## Discussion

Our survey of forest stands impacted by windthrow revealed that this aboveground disturbance had pronounced impact on belowground conditions as well. Consistent with our first hypothesis, soil properties in severely disturbed plots were distinct from those in plots which had not experienced windthrow. The composition of bacterial communities also shifted in response to storm disturbance, confirming our second hypothesis. However, these impacts were felt equally by three different community types: bacterial assemblages in bulk soil, rhizosphere soil, and in association with living roots—contradicting our third hypothesis. Below, we explore the mechanisms by which windstorms might shape biogeochemical and ecological dynamics belowground in the first year following a disturbance event.

### Even mild windthrow disturbances can impact soil biogeochemistry

Less than a year after severe storms, the impacts of windthrow on soil biogeochemistry were still pronounced: affected soils had higher pH, a lower C content, and fewer fine roots. However, these impacts were even more evident in the forest stands that had experienced severe disturbance (involving the formation of pits and mounds due to tree uprooting) *versus* those where the soil was not physically disrupted. Elevated pH in the most disturbed stands could reflect inversion and mixing of soil profiles associated with downed trees, as the soils are shallow peats overlying mineral soil and limestone, and therefore surface soils are more acidic than those at depth. Consistent with this hypothesis, a similar increase in surface soil pH was noted in Welsh forests where soils were mechanically disturbed by tree stump removal (Vanguelova et al. 2017). Meanwhile, the lower C content in severely disturbed stands may also reflect soil profile inversion, and/or accelerated microbial decomposition in those environments (Mayer et al. 2023). Faster decomposition, in turn, could have multiple drivers – higher temperatures due to loss of canopy cover and direct insolation of the soil, higher soil moisture due to loss of canopy and water interception, and/or mechanical disruption of the soil matrix causing the exposure of physically protected carbon. These changes, in turn, could trigger shifts in microbial community structure (discussed further below), causing increased soil respiration and soil carbon leaching. Finally, fine root stocks were substantially lower in the severely impacted stands, suggesting a reduction in the belowground carbon inputs that are the principal source of new soil organic matter formation (Sokol et al. 2019). Interestingly, soil carbon content was significantly elevated in the stands that were only mildly disturbed. This potentially reflects the pulse of leaf litter and coarse woody debris inputs that occurred immediately after the storm (Suzuki et al. 2019). The opposing responses of soil carbon to mild *versus* severe disturbance indicate that multiple processes underlie changes in belowground carbon stocks following a windthrow event. Either gains or losses are possible, depending upon whether the carbon inputs immediately following windthrow outweigh losses due to faster litter decay.

The pronounced changes in fine root abundance observed following windthrow disturbance may provide insight on tree-level responses to environmental change. Few studies have explicitly examined root dynamics in the immediate aftermath of a severe windstorm, although there is ample evidence that subsequent timber salvaging operations may compact the soil and affect root growth (Lüscher 2002). However, canopy gaps – however they are formed – are known to impact patterns of fine root production. In general, in northern temperate forests, small (< 100m^2^) gaps aboveground are mirrored by a decrease in fine root production towards the centre of the gap (Wilczynski and Pickett 1993; Taskinen et al. 2003). In some cases, however, these declines are buffered by increases in the growth of herbaceous species (Campbell et al. 1998). Our findings are generally consistent with prior research, and suggest that the death of fine roots from downed trees is not compensated by a proliferation of new fine roots from the surviving ones on an annual timescale. Interestingly, whereas the loss of canopy cover in moderately and severely wind-disturbed stands was comparable (Fig. 2b**),** the decline in fine roots was more pronounced in the more disturbed stands (Fig. 3**).** This could be an issue of statistical power – there were fewer replicate plots of the intermediate disturbance class – or it could suggest that the extensive physical disturbance to the soil associated with severe windthrow, such as the formation of soil pits and mounds, also plays a role in suppressing root production.

### Soil bacterial communities respond to forest windthrow events

Soil bacterial communities in disturbed stands were compositionally distinct from those in the intact forest plots and tended to be more species-rich, although patterns of ASV evenness did not vary. However, the differences were subtle: no individual phylum, nor any of the most abundant ASVs, exhibited significant abundance variation by disturbance class. This suggests that the biogeochemical perturbations induced by windthrow were not sufficient to substantially alter the phylum-level taxonomic composition of bacterial communities. There have been relatively few investigations of windthrow impacts on soil microbes, but our results are in agreement with a handful of other studies which document weak compositional responses to windthrow and post-storm salvage operations (Gömöryová et al. 2017; Šimonovičová et al. 2019). However, catabolic profiles of microbial communities can exhibit significant shifts even decades after a disturbance event (Osburn et al. 2022) – we do not know if soil bacteria in windthrown stands at Kielder forest are strongly functionally distinct from those in undisturbed stands, e.g. due to physiological shifts in carbon use efficiency or enzyme expression. Additionally, studies of ectomycorrhizal communities, which would dominate at Kielder, post-windthrow revealed reductions in diversity and abundance that can persist for at least decade after the initial disturbance event (Egli et al. 2002).

Differences in the magnitude of microbial responses to windthrow might be due to the nature of the communities examined – the dynamics of mycorrhizal fungal communities are directly influenced by those of their host plants, and they form large, multicellular, perennial networks. By contrast, bulk soil microbiota, composed of individual bacterial cells, may be less affected by tree mortality, but more influenced by soil substrate availabilities and changes. We expected to find that windthrow disturbance would have the strongest impact on root-associated bacteria, which are, presumably, directly impacted by the changes in root growth and turnover we observed. Rhizosphere communities might also be affected, given that an expanding root, as it forages through the soil, shapes the composition of the bacterial communities surrounding it (Bruggen et al. 2000). Yet there was no evidence that bacterial communities in bulk soil, in the rhizosphere, or on roots themselves were differentially sensitive to disturbance.

However, we did find strong differentiation between bacterial communities directly associated with roots (growing on or within the tissue) and those associated with the bulk soil habitat. Several abundant Acidobacteria and Actinobacteria were more prevalent in bulk and rhizosphere soils than on roots, which is expected given these phyla are predominantly associated to the soil habitat. Meanwhile, the reverse was true for Firmicutes, namely various species in order Baciliales. Many bacilli are known to play roles in plant growth promotion, explaining the close association of these taxa to root surfaces (Bloemberg and Lugtenberg 2001).

As a whole, what do these patterns reveal about the mechanisms by which aboveground disturbance impact belowground communities in the short term? In this forest, bacterial beta-diversity was most closely tied to variation in soil carbon and nitrogen, and tended to be influenced by soil pH and root biomass too. All of these factors were directly impacted by windthrow, especially in the stands that were most severely affected by storms. Because these biogeochemical changes appeared to have equal impacts on bulk, rhizosphere, and root-associated microbes, it is likely that bacteria are responding to changes that affect the whole soil profile, such as altered litter inputs, as well as changes in root growth, exudation and turnover.

### Landscape heterogeneity and its interaction with disturbance effects

We observed pronounced heterogeneity in all soil and bacterial community properties across Kielder Forest, with soil carbon content alone varying by 800% among the ten sites. Twenty percent of the variation in bacterial communities could be explained by site identity, although this variation could not be explained by cross-site variation in the soil biogeochemical factors examined (soil carbon, pH, etc.). As the composition of tree communities at Kielder is relatively homogenous, site variation may relate to differences in previous land use, topographic position, or understory flora.

**Fig. 7:**
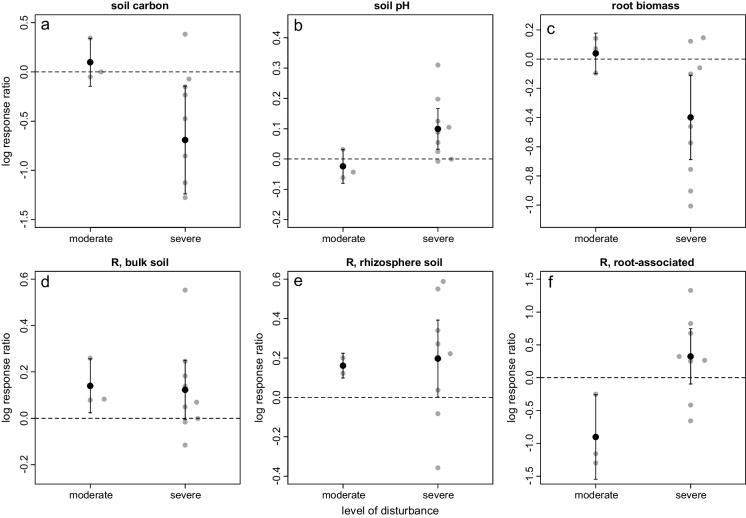
Log response ratios (ln[disturbance/control]) for **a.** soil carbon, **b.** soil pH, **c.** root biomass, and ASV richness (R) in **d.** bulk soil, **e.** rhizosphere soil, and **f.** plant roots. Log response ratios were calculated separately for moderately and severely disturbed plots within each site. Gray points show log response ratios for each individual site, whereas the black points show the mean and 95% confidence interval of each log response ratio across all sites. Note the difference in y-axis scale for panels **d-f**

We used log response ratios to assess the magnitude and direction of the disturbance response for soil carbon, pH, root biomass, and bacterial ASV richness. For the biogeochemical variables, we found that the magnitude of the LRRs tended to vary substantially among sites (Fig. 7), although the *sign* of the LRRs (i.e., whether disturbance increased or decreased a particular variable) was relatively consistent. For microbial variables, treatment responses were more variable in the most disturbed plots, especially for bacterial species richness. Depending upon site identity, either reductions or increases in bacterial diversity were observed in response to severe windthrow (Fig. 7d-f). Stochastic processes are very important in mediating bacterial community assembly following disturbance (Zhang et al. 2016). It is likely that the dramatic biogeochemical changes in severely disturbed plots triggered reorganisations in microbial community structure, which were dominated by random assembly processes (e.g. local extinction, stochastic dispersal). Therefore, although soil bacterial communities did not exhibit consistent compositional shifts in response to windthrow at the landscape scale, disturbance effects were nonetheless quite substantial within some individual sites. It remains to be seen whether these very local disturbance impacts will shape soil function and plant community dynamics in the forest as a whole as it recovers from severe storms.

## Conclusions

Less than a year after severe storms, forest plots that had experienced windthrow exhibited pronounced changes in soil properties. Although bacterial community responses to disturbance were more subtle, they indicate that bacteria respond to altered litter inputs and soil upheaval. Further investigations at Kielder forest will be necessary to reveal the long-term trajectory of forest recovery. However, it is possible that the patchy nature of this disturbance, together with the marked alteration to soil biogeochemistry in severely disturbed plots, will leave a lasting imprint on the spatial dynamics and functioning of this ecosystem.

## Supplementary Information

Below is the link to the electronic supplementary material.

Supplementary file1 (DOCX 14.9 KB)

## Data Availability

Data associated with this manuscript can be accessed at the CEH Environmental Information Data Centre: https://catalogue.ceh.ac.uk/documents/7811769d-3e11-4b79-b781-765ca9cfd83e
