# Supplementary material for: Windthrow disturbance impacts soil biogeochemistry and bacterial communities in a temperate forest
Source: Plant Soil. 2024 Nov 26;512(1-2):395–408. doi: 10.1007/s11104-024-07086-8 (PMC12370869; doi:10.1007/s11104-024-07086-8)
Supplement: Supplementary file 1 — Supplementary file1 (DOCX 14.9 KB) [file 11104_2024_7086_MOESM1_ESM.docx]

**Supplementary Information**

**Appendix 1 – Cano.py**

To extract hemispherical representations of tree canopies from cylindrical (panoramic) photographs in a high-throughput manner, we created cano.py, a python tool based upon the web application Cano.Fi (<https://doi.org/10.5281/zenodo.5171971>) that uses additional tooling from Hemiphot.R (<https://github.com/Hans-ter-Steege/Hemiphot>). The cano.py analysis suite allows for local, parallelized processing and builds upon Cano.Fi to provide additional functionality.

The Cano.py pipeline: 1) automates the process of cropping cylindrical imagery to isolate the canopy from the ground (which is often captured due to the relatively wide angles of phone camera lenses), 2) uses an updated version of Cano.Fi’s code to reproject the cylindrical image onto a hemisphere, 3) applies a threshold to the blue channel of the resultant image (to create a binary map of “sky”) and applies methods adapted from Hemiphot.R to calculate Openness and methods adapted from Cano.Fi to calculate Leaf Area Index (LAI). If cleaning of the projected image is required due to artifacts in the original photograph, this can be completed manually or heuristically before passing the photo to the rest of the analysis pipeline.

The full code and documentation can be found at <https://github.com/fwimp/Cano.py>.
